# *Bordetella pertussis* isolates vary in their interactions with human complement components

**DOI:** 10.1038/s41426-018-0084-3

**Published:** 2018-05-09

**Authors:** Charlotte Brookes, Irene Freire-Martin, Breeze Cavell, Frances Alexander, Stephen Taylor, Ruby Persaud, Norman Fry, Andrew Preston, Dimitri Diavatopoulos, Andrew Gorringe

**Affiliations:** 1 Public Health England, Porton Down, Salisbury, UK; 2 Public Health England, 61 Colindale Avenue, London, UK; 3 Department of Biology and Biochemistry, The Milner Centre for Evolution, University of Bath, Bath, UK; 4 Laboratory of Medical immunology, Nijmegen Medical Centre, Radboud University, Nijmegen, The Netherlands

## Abstract

Whooping cough is a re-emerging respiratory tract infection. It has become clear that there is a need for better understanding of protective immune responses and variation between *Bordetella pertussis* strains to aid the development of improved vaccines. In order to survive in the host, *B. pertussis* has evolved mechanisms to evade complement-mediated killing, including the ability to bind complement-regulatory proteins. Here we evaluate the variation in interactions with the complement system among recently isolated strains. Isolates whose genomes appear highly similar and cluster together on a SNP-based dendrogram were found to vary significantly in resistance to complement-mediated killing and in the deposition of C3b/iC3b, C5b-9 and C1 esterase inhibitor (C1-INH). The key role of Vag8 as a receptor for C1-INH was confirmed and its expression was shown to vary in a panel of isolates. A Vag8 knockout mutant showed increased sensitivity to complement-mediated killing. Antibodies in convalescent sera blocked C1-INH binding to *B. pertussis* and may play an important role in natural immunity.

## Introduction

*Bordetella pertussis* is the causative agent of whooping cough, a serious respiratory infectious disease in all age groups, with young infants at the greatest risk of severe disease. Despite high vaccine coverage, there has been an increase in the incidence of disease in the UK, Europe, Australia and in the US over the past 10 years^1^ and whooping cough is now the most prevalent ‘vaccine preventable’ disease in these countries. The UK observed an increase in laboratory-confirmed pertussis cases from 2011 and experienced 9741 cases in 2012 with 14 infant deaths. This led to the introduction of maternal vaccination with an acellular pertussis (aP) vaccine, which has been effective in preventing severe disease in the very young^2^. The resurgence of pertussis is likely to be due to a combination of factors including faster waning immunity following aP vaccination in comparison to whole-cell (wP) vaccinated individuals^3^ and strain evolution^4^. In addition, the type of the immunity induced by aP vaccination has been found to differ to immunity induced by the wP pertussis vaccination^5^. Evidence from the baboon model of pertussis disease suggests that aP vaccination may be less effective at providing clearance of colonisation, whereas wP vaccination can induce immunity that interferes with and significantly reduces colonisation^6^. It has become clear that there is a need for better understanding of the pathogenesis of *B. pertussis*, protective immune responses and variation between strains to aid the development of improved vaccines.

The complement system is a key component for defence against pathogenic microorganisms that invade the bloodstream or mucosal surfaces. Intact mucosal surfaces have about 10% of the complement concentration of serum and the amount increases during infection^7^. Thus evasion of this system is a survival strategy employed by many bacterial pathogens^8^. Complement activation can occur through the classical pathway (CP), lectin pathway (LP) or the alternative pathway (AP) and induces opsonic, lytic and inflammatory responses. The CP is initiated when antibody and antigen complexes interact with the C1 complex, leading to cleavage of C4 and C2 and the formation of the C3 convertase (C4b2a). The LP is initiated via mannose-binding lectin (MBL) or ficolins forming a complex with the mannose-binding lectin-associated serine proteases on the pathogen surface and also leads to the formation of the C3 convertase (C4b2a). AP activation involves continuous hydrolysis of C3 leading to the formation of the alternative C3 convertase (C3bBb) through binding with factor B and the action of factor D. The AP is an amplification loop for the CP and LP. The C3 convertases cleave C3 and the generated C3b deposits onto the bacterial membrane where it can mediate opsonophagocytosis and the formation of the C5 convertase. Cleavage of C5 leads to the deposition of C5a and then the formation of the membrane attack complex, which is important in mediating killing. It has been proposed that opsonisation, phagocytosis and complement-mediated killing is important for protection against pertussis^9^. Antibody and complement-mediated opsonophagocytosis of *B. pertussis* has been demonstrated using sera from individuals with evidence of recent infection^10^ and following vaccination^11, 12^. Antibody and complement-mediated bactericidal activity has also been demonstrated in serum from infected adults^13^ but bactericidal activity was not increased following aP vaccination^13, 14^. The importance of T helper type 1 (Th1)/Th17 immune responses for protection against pertussis is now clear^15^, facilitating the induction of opsonising antibodies and recruiting and activating neutrophils. Antibody and complement interactions with *B. pertussis* are an important part of this picture and one that requires further characterisation.

Pathogenic bacteria can avoid complement-mediated killing by utilising regulatory proteins of the complement cascade, and many of these have been shown to be protective vaccine antigens. *B. pertussis* has been reported to use *Bordetella* resistance to killing antigen (BrkA)^16^, filamentous haemagglutinin (FHA)^17^, autotransporter virulence-associated gene 8 (Vag8)^18^ and *B. pertussis* autotransporter protein C^19^ to evade complement-mediated killing. It has been shown that BrkA can reduce the deposition of C3 and C4 on the surface of *B. pertussis* thus reducing membrane attack complex formation, although the mechanism for this is still not known^16^. C1-esterase inhibitor (C1-INH) has been shown to bind Vag8 expressed by *B. pertussis*^16^. C1-INH is a negative regulator of CP and LP activation. Vag8 on an outer membrane vesicle (OMV) or as a secreted passenger can bind C1-INH and lead to cleavage of C4 and C2 by proteases and result in depletion in the liquid phase of these components, inhibiting CP and LP activation^20^. It has been demonstrated that FHA binds C4b-binding protein (C4BP), inhibiting both the classical and lectin pathways of complement activation^17^. *B. pertussis* has also been shown to bind Factor H (FH), which is a negative regulator of the alternative pathway^21^. In addition to *B. pertussis* using complement-regulatory proteins to evade complement-mediated killing, it has been reported that there is an increase in the risk of pertussis in patients with an MBL deficiency^22^. The evolution of complement-evasion mechanisms and the increased risk of pertussis in patients deficient in MBL suggests an important role for complement, together with specific antibodies, in protection from pertussis.

This study investigates for the first time whether there is variation among recently circulating *B. pertussis* isolates regarding their interactions with the complement system. Using antibody-depleted human plasma as a complement source, we evaluated the susceptibility of *B. pertussis* to complement-mediated bactericidal killing in the absence of antibodies and compared the deposition of complement components between strains. We have also investigated the role of Vag8 expression and how this determines complement interactions with *B. pertussis* isolates.

## Results

### Variation in survival in IgG-depleted human plasma

**Fig. 1:**
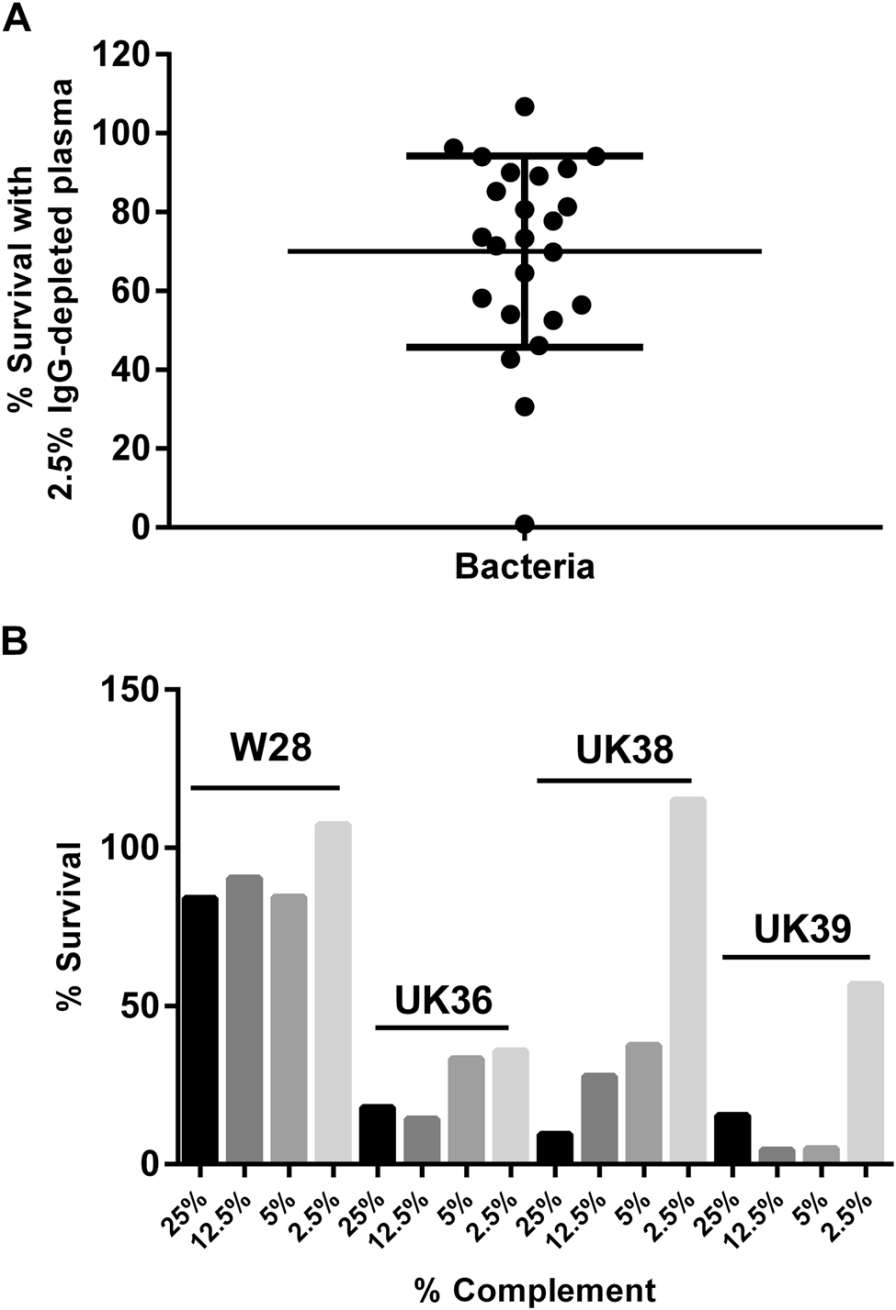
**a** Percentage of survival of *B. pertussis* strains when incubated with 2.5% IgG-depleted human plasma for 1 h. Bars = mean and standard deviation. **b** Percentage of survival of *B. pertussis* strains, Wellcome 28 (W28), UK36, UK38 and UK39, following incubation with either 25, 12.5, 5 or 2.5% IgG-depleted human plasma for 1 h

A panel of 24 UK strains was selected from 100 genome-sequenced isolates^23^ to include strains that were diverse and strains that were found to cluster together on a single-nucleotide polymorphism (SNP)-based dendrogram from the 2012 outbreak in the UK. A number of isolates from before 2012, whole-cell vaccine strain Wellcome 28, Tohama I and a recent isolate from the Netherlands, B1917^24^ were also included. The UK 2012 isolates clustered with strains isolated during the early 2000s from a variety of geographical areas, including North America, Europe and Australia^23^. The isolates were incubated for 1 h in 2.5% immunoglobulin G (IgG)-depleted human plasma and survival was determined. We observed a large variation between strains in their sensitivity to complement-mediated killing (Fig. 1a), with survival ranging from 1 to 106%. The differences in sensitivity to antibody-independent bactericidal killing were further characterised for strain Wellcome 28 and three UK 2012 isolates that were isolated in the same geographical region and that clustered together on a SNP-based dendrogram^23^. Strain Wellcome 28 demonstrated a high level of resistance to complement-mediated killing while UK isolates UK36, 38 and 39 were found to be more sensitive to complement killing (Fig. 1b). It was striking that while 36 and 57% survival was observed for UK 36 and 39, respectively, no killing was seen with UK38. Information on whether the patient was hospitalised or died was available for some of the isolates (Table 1), but the numbers were insufficient to determine whether increased survival in IgG-depleted human plasma was associated with increased virulence.

**Table 1:** Characteristics of the strains used in this study

Strain no.	Age	Admitted to hospital/fatal case	Year of isolation	Serotype	*ptxP*	*ptxA*	*prn*	Reference
Tohama I	NK	NK/NK	1954	1, 2	1	2	1	^24^
W28	NK	NK/NK	NK	1, 2, 3	ND	ND	ND	^41^
B1917	44 months	NK/NK	2000	1, 3	3	1	2	^26^
UK3	NK	NK	1946	1, 2, 3	1	2	1	^24^
UK4	NK	NK	1947	1, 2	1	1	1	^24^
UK5	NK	NK	1949	1, 3	1	2	1	^24^
UK6	NK	NK	1967	1, 2, 3	1	1	1	^24^
UK9	NK	NK	1982	1, 2	1	1	3	^24^
UK10	NK	NK	1982	1, 2, 3	1	1	1	^24^
UK11	NK	NK	1983	1, 3	1	1	1	^24^
UK25	3 months	Yes/No	2008	1, 3	3	1	2	^24^
UK35	1 months	Yes/Yes	2012	1, 2	3	1	2	^24^
UK36	15 years	No/No	2012	1, 3	3	1	2	^24^
UK38	14 years	No/No	2012	1, 3	3	1	2	^24^
UK39	16 years	No/No	2012	1, 3	3	1	2	^24^
UK54	16 years 2 months	Yes/NK	2011	1, 3	3	1	2	^24^
UK58	1 months	Yes/No	2012	1, 2	3	1	2	^24^
UK61	12 years 1 months	Yes/No	2012	1, 3	3	1	2	^24^
UK63	64 years 6 months	No/No	2012	1, 3	3	1	ND	^24^
UK67	3 months	Yes/No	2012	1, 2	3	1	2	^24^
UK69	14 years 10 months	No/No	2012	1, 3	3	1	2	^24^
UK70	2 months	Yes/No	2012	1, 3	3	1	2	^24^
UK72	27 years 3 months	No/No	2012	1, 3	3	1	2	^24^
UK73	1 months	Yes/NK	2012	1, 2	3	1	2	^24^
UK75	3 months	Yes/No	2012	1, 3	3	1	2	^24^

*NK* not known, *ND* not determined

### Variation in antibody-independent complement component deposition onto a panel of *B. pertussis* strains

**Fig. 2:**
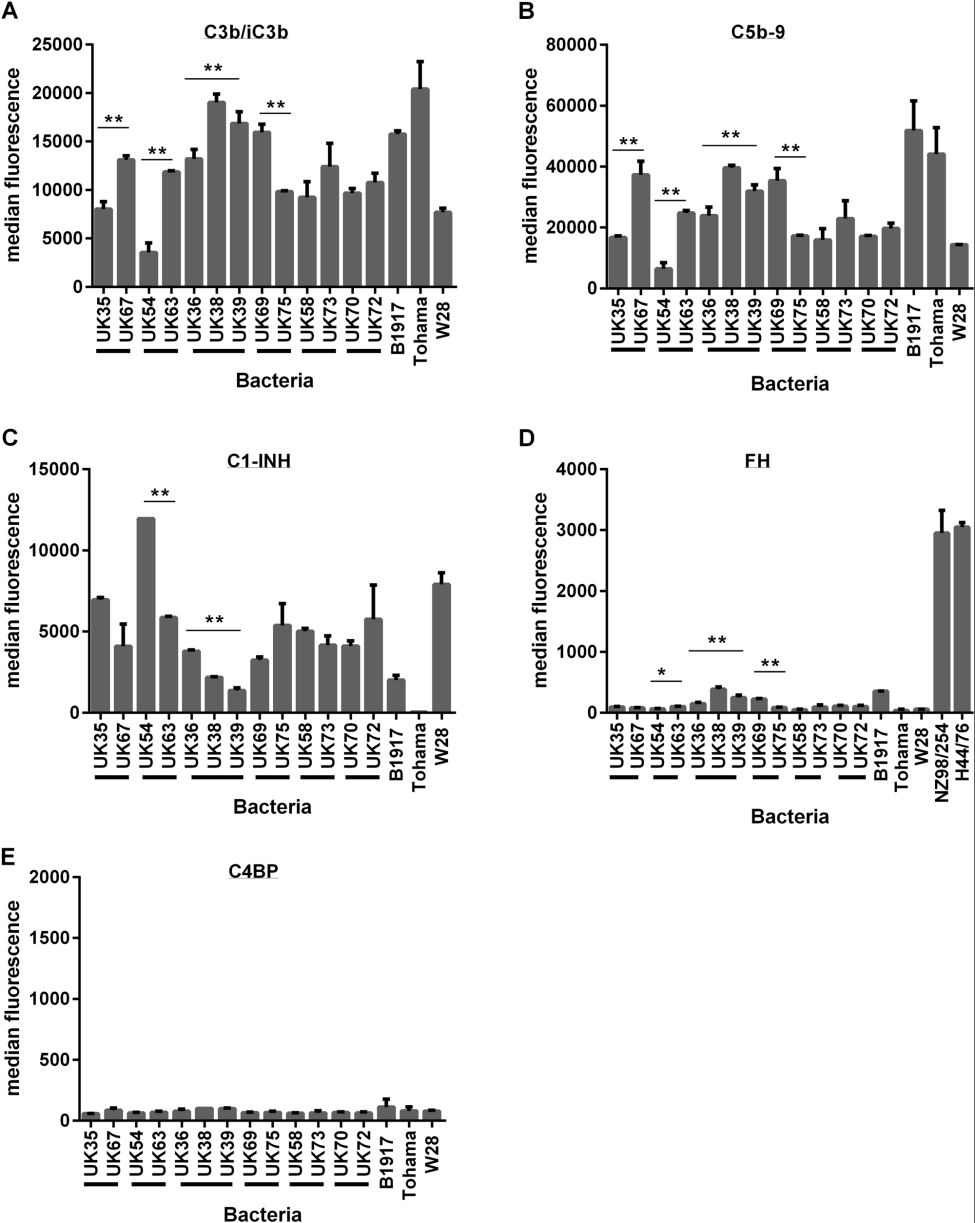
Antibody-independent deposition of complement components **a** C3b/iC3b, **b** C5b-9, **c** C1-INH, **d** FH and **e** C4BP onto a group of *B. pertussis* isolates (*n* = 16). Each value is the mean of two determinations and the error bar is the standard deviation. Black bars below the *x* axis indicate groups of isolates characterised as very closely related and clustering on a SNP-based dendrogram (**p < 0.001 and *p < 0.05 by *T*-test)

**Fig. 3:**
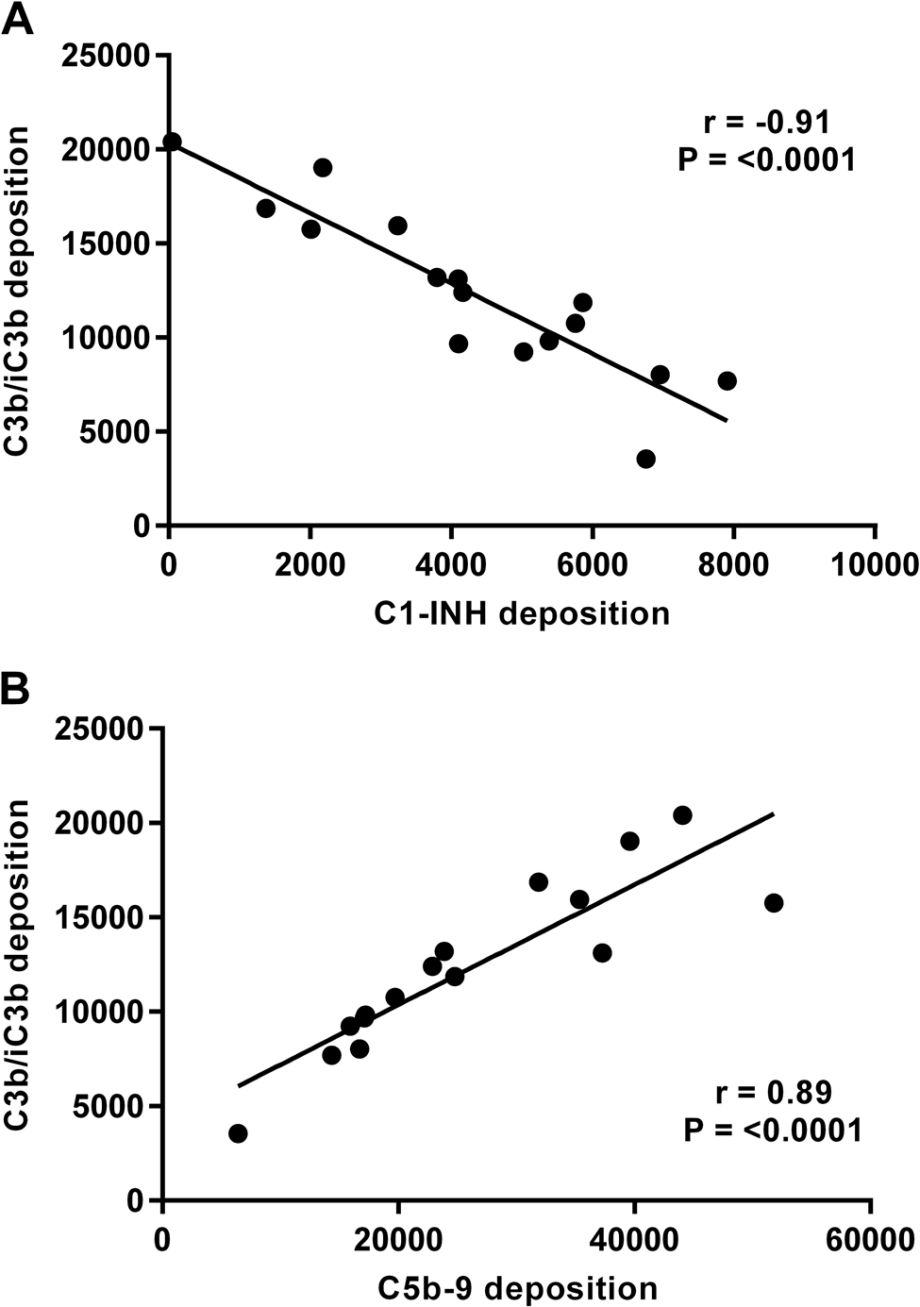
**a** Correlation of antibody-independent complement deposition of C3b/iC3b and C1-INH onto a group of *B. pertussis* isolates, *n* = 16 (*r* = −0.91, *p* < 0.001). **b** Correlation of antibody-independent complement deposition of C3b/iC3b and C5b-9 onto a group of *B. pertussis* isolates *n* = 16 (*r* = 0.88, *p* < 0.001)

A smaller panel of isolates was selected (*n* = 16 to include groups of 2012 isolates that were either characterised as SNP divergent or found to be closely related and identical by SNP analysis (Table 1)). Isolates were incubated with 2.5% IgG-depleted human plasma and then antibody-independent deposition of complement components C3b/iC3b, C5b-9, C1-INH, FH and C4BP was evaluated by flow cytometry. This was compared with binding of these components to strains B1917, Tohama I and Wellcome 28. There were significant differences in the levels of deposition of components C3b/iC3b, C5b-9 and C1-INH for isogenic strains UK54 and 63 and also for UK36, 38 and 39 (*p* < 0.01). Significant differences were also observed between UK35 and 67 and UK69 and 75 in the antibody-independent deposition of C3b/iC3b and C5b-9 (Fig. 2a, b). *B. pertussis* B1917, Tohama I and Wellcome 28 also showed differences in the levels of complement components deposited. Tohama I demonstrated the lowest levels of C1-INH deposition observed for this panel of strains and high levels of C3b/iC3b and C5b-9 binding (Fig. 2a–c). High levels of C1-INH deposition were seen on Wellcome 28 together with lower levels of C3b/iC3b and C5b-9. B1917, a recent *ptxP3* strain^25^, showed deposition levels similar to other strains evaluated (Fig. 2). Antibody-independent deposition of FH was evaluated with this panel of *B. pertussis* strains and was compared with the binding of FH by *Neisseria meningitidis* strains NZ98/254 and H44/76 as control positive organisms. *N. meningitidis* has been shown to bind FH via the FH-binding protein and is able to use this as a complement-regulating mechanism and was thus included as a positive control^26^. Significant differences were observed between UK54 and 63, between UK68 and 75 and also for UK36, 38 and 39 (*p* > 0.001). However, the overall deposition of FH binding by *B. pertussis* was low in comparison with *N. meningitidis* (Fig. 2d). Antibody-independent deposition of C4BP was evaluated with low levels observed across all the strains tested with no significant differences seen between the strains (Fig. 2e). There was a strong negative correlation between the antibody-independent deposition of C3b/iC3b onto the *B. pertussis* isolates and the binding of C1-INH (*r* = −0.91, *p* < 0.001). Highest levels of C3b/iC3b deposition were seen in the strain that bound the lowest levels of C1-INH (Fig. 3a). In addition, antibody-independent deposition of C3b/iC3b was found to strongly positively correlate with C5b-9 deposition (*r* = 0.88, *p* < 0.001) (Fig. 3b).

### Complement-mediated killing correlates with the deposition of complement components

**Fig. 4:**
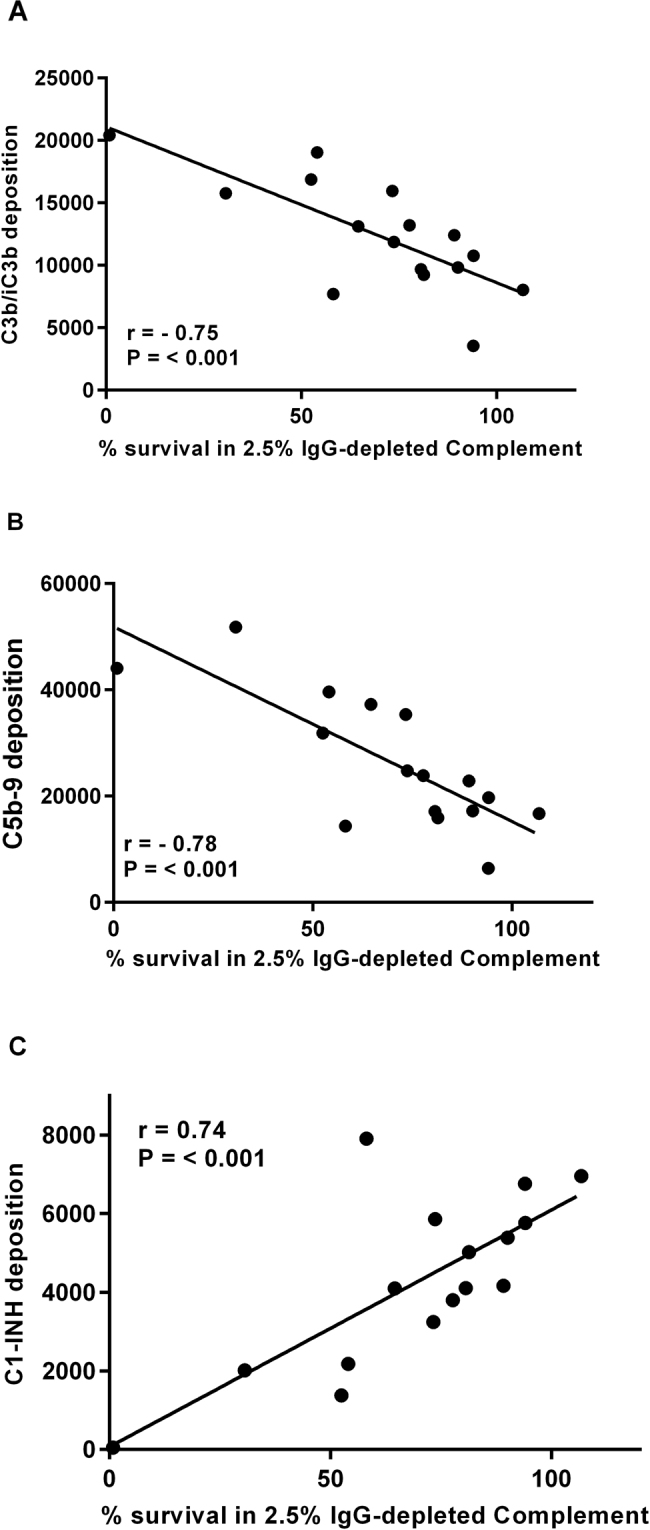
Correlation of antibody-independent deposition of C3b/iC3b (*r* = −0.74, *p* < 0.001) (**a**), C5b-9 (*r* = −0.78, *p* < 0.001) (**b**) and C1-INH (*r* = 0.74 *p* < 0.05 (**c**) with percentage of survival following incubation with 2.5% IgG-depleted human plasma on a group of *B. pertussis* isolates (*n* = 16)

*B. pertussis* isolates were incubated with 2.5% IgG-depleted human complement for 1 h and percentage of survival was calculated by comparing to a heat-inactivated complement control. Percentage of survival was correlated with the antibody-independent deposition of C3b/iC3b, C5b-9 and C1-INH. C3b/iC3b and C5b-9 deposition correlated negatively with percentage of survival of isolates (*r* = −0.74, *p* < 0.001 and *r* = −0.78, *p* < 0.001) (Fig. 4a, b). Highest levels of C3b/iC3b and C5b-9 were found on the strains with the lowest percentage of survival when incubated with 2.5% IgG-depleted human complement. Antibody-independent C1-INH deposition was found to positively correlate with the percentage of survival of isolates following incubation with 2.5% IgG-depleted human complement for 1 h (*r* = 0.74, *p* < 0.05) (Fig. 4c), with the highest levels of C1-INH deposition found on strains that showed the greatest percentage of survival.

### Variation in Vag8 expression correlates with C1-INH binding

**Fig. 5:**
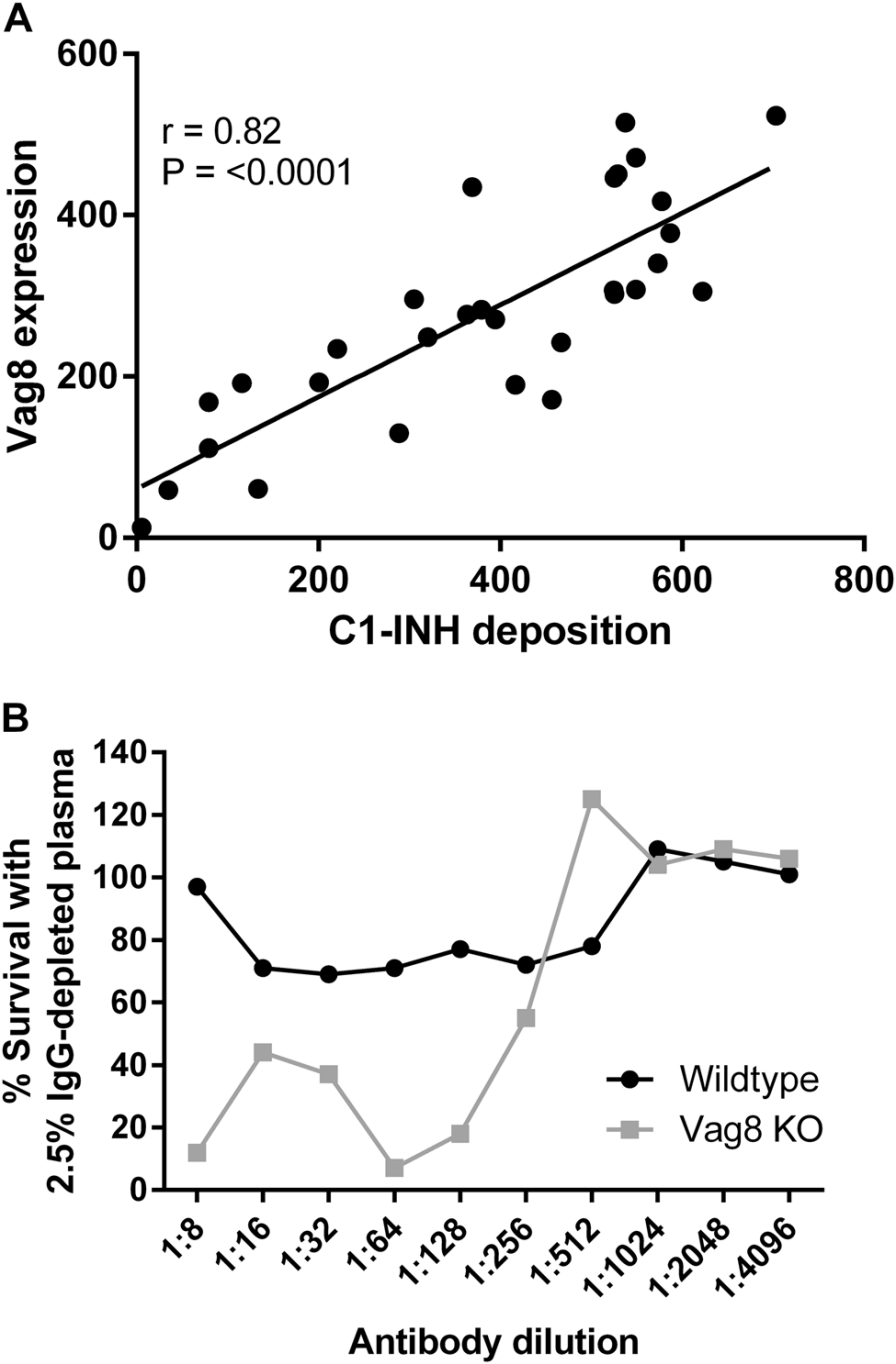
**a** Vag8 expression correlated with the deposition of C1-INH on a panel of *B. pertussis* isolates *n* = 30 (*r* = 0.82, *p* < 0.001). **b** Percentage of survival of *B. pertussis* B1917 WT and Vag8 knockout mutant in 2.5% IgG-depleted human plasma and dilutions of NIBSC 06/140 human serum compared with the no antibody control

The expression of the surface antigen Vag8, the receptor for C1-INH, was determined by flow cytometry using mouse antiserum raised against recombinant Vag8. Vag8 expression in the isolates tested was found to vary greatly, with the X-median fluorescence ranging between 2.47 and 631.1 (Fig. 5a). Antibody-independent C1-INH deposition was found to positively correlate with Vag8 expression (*r* = 0.82, *p* < 0.001), strongly supporting a key role for Vag8 in the binding of this complement component. Serum bactericidal activity was performed to compare the killing of wild-type *B. pertussis* B1917 with a B1917 *vag8* knockout (B1917Δ*vag8*). This was done using dilutions of World Health Organisation (WHO) International Standard Pertussis antiserum (NIBSC 06/140). B1917Δ*vag8* was found to be significantly more susceptible to antibody and complement-mediated killing than the B1917 *B. pertussis* wild type (Fig. 5b).

### Antibodies in convalescent sera block C1-INH binding

**Fig. 6:**
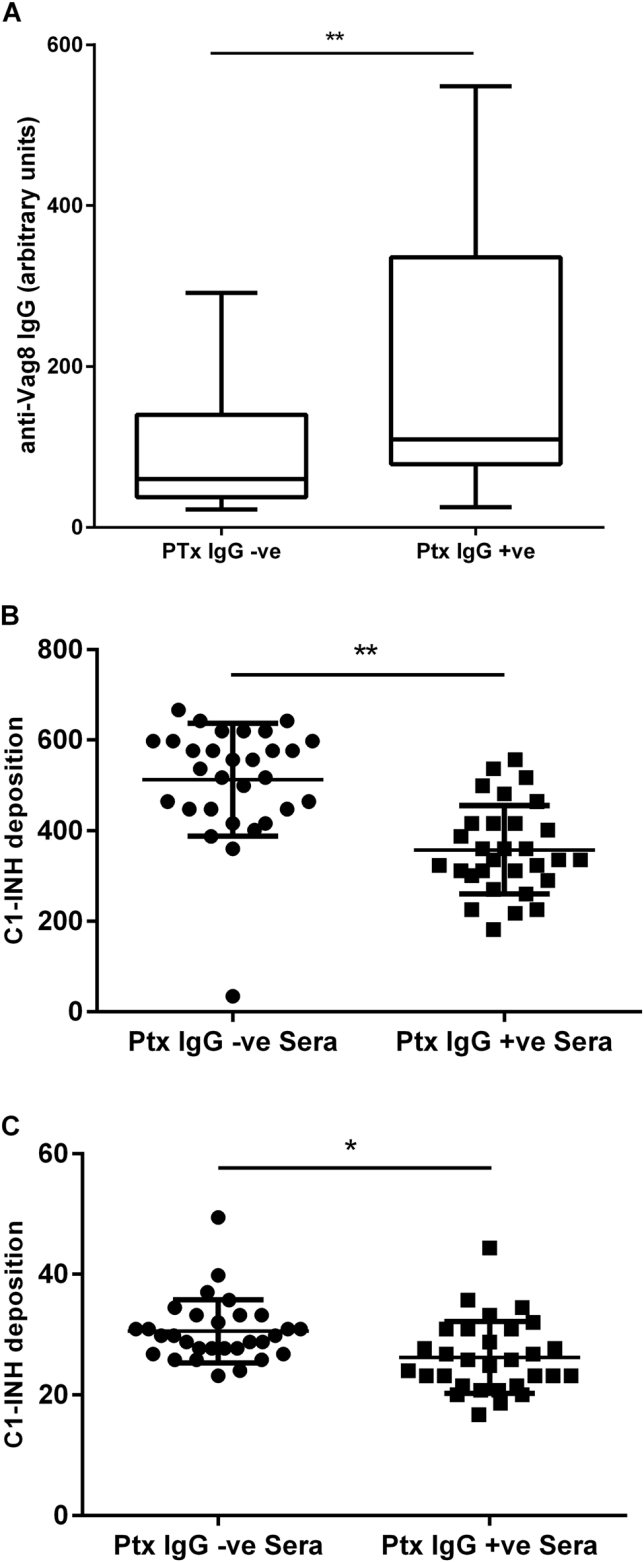
**a** ELISA measuring Vag8-specific antibody in 60 Ptx IgG-negative non-convalescent and Ptx IgG-positive (convalescent) sera (**Mann–Whitney test *p* < 0.01). **b** Effect on C1-INH deposition following pre-incubation of *B. pertussis* UK61 (high Vag8-expressing strain) or UK25 (low Vag8-expressing strain) with either Ptx IgG-positive convalescent sera (*n* = 30) or Ptx IgG-negative non-convalescent sera (*n* = 30) (**p < 0.001 and *p < 0.05 by T-test)

Using an enzyme-linked immunosorbent assay (ELISA) to measure Vag8-specific antibody, significantly higher anti-Vag8 IgG concentrations were found in anti-Ptx IgG-positive convalescent sera in comparison with anti-Ptx IgG-negative sera (Fig. 6a). We also assessed the ability of sera to block C1-INH binding to *B. pertussis* using two target strains that had been identified as expressing either high (UK61) or low (UK25) levels of Vag8 expression. Anti-Ptx IgG-positive convalescent sera significantly reduced C1-INH binding to UK61 (*p* < 0.001) and UK25 (*p* < 0.05) in comparison to anti-Ptx IgG-negative sera (Fig. 6b, c), demonstrating that *B. pertussis* infection-induced antibodies are able to block Vag8-dependent C1-INH binding.

## Discussion

There has been a resurgence of pertussis in many countries despite high vaccination coverage^27^. This has been attributed to a combination of *B. pertussis* strain evolution, the different immune response elicited and faster waning immunity provided by aP vaccines^4, 28–31^. There is also evidence from the baboon model of pertussis that acellular vaccines may be less effective in reducing colonisation than whole-cell vaccines^6^. Development of improved vaccines has now become important and immunoassays to measure functional antibody responses and complement interactions with *B. pertussis* are likely to be important to evaluate future vaccine candidates. Further understanding of interactions of complement proteins with *B. pertussis* can also be used to identify differences in strain fitness to cause infection and disease. This will also aid the selection of representative strains for use in immunoassays to assess natural and vaccine-induced immunity. In addition, understanding the role of complement-regulatory proteins will inform on their potential as future vaccine candidates.

Here we have evaluated the interactions of *B. pertussis* isolates with IgG-depleted human plasma as a source of complement, comparing the differences between circulating strains, evaluating the role of Vag8 in binding complement-regulatory protein C1-INH and the effect of this on complement deposition and survival of isolates. *B. pertussis* isolates were selected for analysis based on a SNP-based dendrogram, including strains clustering closely together and strains that did not cluster^23^. We observed significant differences between isolates in their survival in IgG-depleted human plasma. Unexpectedly, several strains with identical SNP-based genotypes demonstrated significant differences in their susceptibility to complement-mediated killing. Significant differences were also observed in the antibody-independent binding of complement components C3b/iC3b, C5b-9, C1-INH and FH to isolates that were very closely related by genomic analysis. These differences were surprising as it was expected that they would behave similarly in their interactions with complement. The *B. pertussis* genome has been shown to be highly conserved among strains in both a study investigating 343 isolates from around the world^25^ and a study investigating the variation among UK outbreak isolates^23^. Our findings challenge the assumption that strains whose genomes appear highly similar when short sequence reads are mapped onto a reference genome will have the same phenotype. A potential explanation for the differences between genotype and phenotype may be due to differences in genome arrangement, which was not evaluated for this panel of isolates as genome assembly is not possible with Illumina short sequencing reads due to multiple copies of insertion sequences. All the isolates were cultured in conditions that allow the global regulatory BvgA/S system to express Bvg-regulated genes, including Vag8. Thus this observed variability of Vag8 expression suggests other factors that affect expression. One of these factors may be growth phase as it has been shown that *B. pertussis* in log-phase liquid culture were more sensitive to complement killing than bacteria from stationary-phase cultures^32^. It was noted in the current study that Tohama I and Wellcome 28 reached a higher culture density following 24 h incubation in liquid medium than the more recent isolates. The role of growth phase in expression of complement resistance antigens by *B. pertussis* should be carefully determined in future studies.

C4BP is an inhibitor of the CP and LP and acts by interfering with the formation of C4bC2a. *Streptococcus pyogenes*^33^, *Haemophilus influenzae*^34^, *Neisseria meningitidis*^35^ and *Escherichia coli*^36^ have all been found to recruit C4BP to escape complement-mediated killing. C4BP has also previously been found to bind to the surface of *B. pertussis*, primarily via FHA^17^. However, it is unclear what role C4BP binding has in resistance to complement killing as FHA mutants have been shown to be equally resistant to complement killing in comparison with a wild-type strain^37^. In this study, low of levels of C4BP binding was observed for all strains in the tested panel, suggesting that C4BP binding may be less important for the regulation of complement interactions with *B. pertussis* than other components such as C1-INH, at least under the conditions tested. FH is a regulator of complement that is bound by *N. meningitidis*. The meningococcal FH-binding protein is a component of the licensed vaccine Bexsero^38^ and induces bactericidal antibody against meningococcal isolates with a matched FHbp antigen. FH has been shown to bind to *B. pertussis* and to be able to regulate complement activation^21^. However, in this study low levels of FH deposition were measured on the *B. pertussis* isolates assessed and FH binding was ten-fold lower than seen with *N. meningitidis* H44/76 and NZ98/254 strains. This suggests a less important role in regulation of complement on *B. pertussis* than for *N. meningitidis*.

Tohama I and Wellcome 28 strains were included in these studies to compare how these strains differ in their interaction with complement components to more recently isolated UK strains and the Dutch isolate B1917^25^, which has been selected for use in a human challenge model^39^. Tohama I, a strain isolated in Japan in the 1950s and widely used in vaccine manufacture, and Wellcome 28^40^, a whole-cell vaccine strain, both do not express the type III secretion system effector Bsp22^41^. This type III secretion system subverts innate and adaptive immune responses and its absence has raised concerns about the use of these strains as representative targets for functional antibody immunoassays. In this study, we present further evidence that supports these concerns. Tohama I and Wellcome 28 demonstrated distinct binding patterns with complement factors compared to the more recent isolates. Tohama I demonstrated very low levels of C1-INH, FH and C4BP binding, but very high levels of C3b/iC3b and C5b-9 deposition, as well as a high susceptibility to complement killing. Wellcome 28 showed high levels of C1-INH and low FH, C4BP binding, with low C3b/iC3b and C5b-9 deposition. Wellcome 28 was also one of the most resistant strains to bactericidal killing. The strain-to-strain variability in binding of complement components C3b/iC3b, C5b-9, C1-INH, FH and C4BP emphasise the importance of careful strain selection to measure complement-mediated functional antibody activity.

This study confirms the key role of Vag8 as a receptor for C1-INH and demonstrates that strain variation in Vag8 expression impacts on binding of human complement proteins and resistance to complement-mediated killing. Understanding how and why the regulation of Vag8 expression differs between isolates that have the same genotype may shed light on the resurgence of pertussis and is the subject of ongoing investigation. Vag8 expression by *B. pertussis* facilitates C1-INH binding and is used as a complement evasion strategy. It has been suggested that *B. pertussis* may be able to bind C1-INH onto secreted OMVs expressing Vag8 or as secreted Vag8^20, 42^. Without C1-INH present, C4 and C2 are cleaved by proteases leading to their depletion in the liquid phase and a reduced ability to form the CP and LP C3 convertases on the bacterial surface^20^. This study focusses on the effect of surface-bound Vag8 and C1-INH interactions and shows that levels of surface-bound C1-INH correlate with reduced deposition of C3b/iC3b and C5b-9 and allows the bacteria to survive in human antibody-depleted complement. Thus surface-bound Vag8 is likely to also play an important role in evasion of complement-mediated killing by *B. pertussis*.

Survival of bacteria following incubation with IgG-depleted complement negatively correlated with the antibody-independent deposition of C3b/iC3b and C5b-9. As expected, high levels of complement deposition on the surface of the bacteria correlated with a low percentage of bacterial survival. C1-INH binding correlated significantly, with inhibition of the classical and lectin pathways of complement activation and increased bacterial survival.

In this study, we show that strain-dependent variation in Vag8 expression on the surface of *B. pertussis* correlated with antibody-independent deposition of C1-INH, which also correlated negatively with survival in IgG-depleted human plasma. In addition to this, a Vag8 knockout strain showed increased sensitivity to antibody-dependent complement-mediated killing in a bactericidal assay in comparison with the wild-type strain. In addition, convalescent sera had significantly higher levels of anti-Vag8 antibodies in comparison to anti-Ptx-negative sera, indicating that, following infection, antibodies are induced that bind Vag8. Induction of anti-Vag8 antibodies has previously been shown following vaccination with wP or OMVs^43^ and following infection. In addition to this, others have shown that vaccination with rVag8 significantly reduce bacterial load in mouse lungs^44^. Using high and low Vag8-expressing isolates, we have shown that antibodies in convalescent sera block C1-INH binding to *B. pertussis*, pointing to a role in natural immunity. Vag8 has been suggested as a potential vaccine antigen^44, 45^ and the data in this study support this suggestion. However, it is important to consider that some strains express very low levels of Vag8. Vag8 in combination with multiple other antigens may thus represent a more attractive vaccine strategy.

Previous studies have characterised the interactions of *B. pertussis* with complement and have reported contrasting findings that maybe the result of variation in the source of complement used and the growth conditions of the strains tested^21, 32, 37, 46, 47^. This study has shown that *B. pertussis* isolates with an identical SNP-based genotype show distinct phenotypes regarding their interactions with complement deposition and survival following incubation with IgG-depleted human plasma. These phenotypic differences between strains are an important observation which changes the assumption that strains whose genomes appear highly similar will have the same phenotype. This study has confirmed the role of Vag8 as the receptor of C1-INH and suggests a role in natural immunity for antibodies that block C1-INH binding, increasing susceptibility to complement-mediated killing.

## Material and methods

### Bacteria

*B. pertussis* isolates used in this study are as described in Table 1. Bacteria were stored at −70 °C in CL-CD medium^48^ containing 10% glycerol. Bacteria were cultured on charcoal agar with sheep blood (Oxoid) incubated for 48 h at 35 °C. Bacteria were then cultured for 24 h at 35 °C in CL-CD medium with orbital shaking.

### Sera

Anonymised residual sera sent to the Public Health England Pertussis Reference Laboratory for serodiagnosis of pertussis following prolonged cough were obtained. These included 30 sera that were seropositive and 30 sera that were seronegative for recent pertussis by anti-Ptx IgG ELISA^49^. Mouse serum was raised against recombinant Vag8^44^ in a group of 5 BalbC mice, which were immunised on days 0, 21 and 28 with blood obtained at day 35.

### Bactericidal assay

Bacteria were resuspended in bactericidal buffer (Hanks buffered saline solution (Invitrogen), 0.5% bovine serum albumin (BSA)) and the OD_600 nm_ was measured. A suspension at an OD_600 nm_ of 1 that contains 2 × 10^9^ bacteria/ml was diluted in bactericidal buffer to 6 × 10^4^ colony-forming units/ml. For each isolate, 10 µl of this suspension was added to 20 µl of bactericidal buffer, to which 10 µl of either IgG-depleted human plasma^50^ or heat-inactivated IgG-depleted plasma was added to give a final concentration of 2.5% in each well of the microplate. The plate was then incubated at 37 °C for 1 h with shaking at 900 rpm. In all, 10 µl was then plated out onto charcoal agar using the tilt method, air dried and incubated at 35 °C for 4 days_._ Following incubation, colonies were counted and percentage of survival was calculated relative to the heat-inactivated complement control. To determine serum bactericidal activity, 20 µl of two-fold dilutions of heat-inactivated test antiserum were added to each well together with the bacteria and 2.5% final concentration of IgG-depleted plasma. Following 1 h incubation at 37 °C with shaking at 900 rpm, 10 µl from each well was plated as described above. Following a 96 h incubation on charcoal agar plates, colonies were counted and a titre assigned to the reciprocal dilution that gave >50% killing compared with the bacteria and complement only control.

### Measurement of antibody-independent C5b-9, C3b/iC3b, C1-INH, FH or C4BP deposition

C1-INH, FH or C4BP binding to live *B. pertussis* was measured by flow cytometry in separate assays while C5b-9 and C3b/iC3b deposition were measured in a duplexed assay. For each assay, 10 µl of IgG-depleted human plasma and 90 µl of target bacteria at an OD_600 nm_ 0.1 in phosphate-buffered saline (PBS) and 1% BSA (blocking buffer (BB)) were incubated for 45 min with shaking (900 rpm) at 37 °C. The samples were then centrifuged at 3060 × *g* for 5 min and washed with BB. This was repeated twice before being resuspended in 200 µl of either anti-human C3b/iC3b-FITC (Abcam, UK) at 1:500 and anti-human SC5b-9-Alexa Fluor 647 nm (Quidel, USA) at 1:4000 or mouse anti-human C1-INH (Fitzgerald, UK) at 1:1000, mouse anti-human FH (Quidel, USA) at 1:200 or mouse anti-human C4BP (Stratech Scientific, UK) at 1:500 in BB. Following 20 min incubation at 4 °C, samples were centrifuged and washed with BB twice more as described above. Assays measuring C1-INH, FH or C4BP then required an additional step of resuspension with 200 µl goat anti-mouse IgG-FITC (Abcam, UK) at 1:500 in BB. This was incubated for 20 min at 4 °C before being centrifuged and washed twice more with BB. The samples were then analysed by flow cytometry.

### Blocking of C1-INH deposition by patient sera

In all, 5 µl of patient serum was incubated with 90 µl of *B. pertussis* at an OD_600 nm_ 0.1 in BB for 45 min with shaking (900 rpm) at 37 °C. Samples were then centrifuged at 3060 × *g* for 5 min and washed with BB twice before resuspension in 90 µl of BB and 10 µl of IgG-depleted human plasma and incubated for 45 min with shaking (900 rpm) at 37 °C. The plate was then centrifuged and washed two times with BB as described above before resuspending with mouse anti-human C1-INH (Fitzgerald, UK) at 1:1000 in BB. Following 20 min incubation at 4 °C, samples were centrifuged and washed twice more with BB before finally resuspending with 200 µl goat anti-mouse IgG-FITC (Abcam, UK) at 1:500 in BB. Samples were incubated for 20 min at 4 °C, centrifuged and washed twice with BB, and the samples were analysed by flow cytometry.

### Vag8 expression

Mouse anti-Vag8 serum (2 µl) at a 1:500 dilution in BB was added to 198 µl of *B. pertussis* at an OD_600 nm_ of 0.1 in BB and incubated for 30 min with shaking (900 rpm) at 25 °C. The samples were then centrifuged at 3050 × *g* for 5 min, the supernatant was removed and the pellet was washed with 200 µl of BB. This was repeated twice before resuspending in goat anti-mouse-FITC conjugate 1:500 in BB. Following incubation for 20 min at 4 °C, the samples were centrifuged and washed twice more with BB. The samples were then analysed by flow cytometry.

### Flow cytometry

Samples were analysed using a LSR Fortessa (Becton Dickenson) flow cytometer. Bacteria were identified based on their forward scatter and side scatter. The samples were evaluated for fluorescence of the whole population, and each test was the average median fluorescence of duplicate tests.

### Anti-Vag8 IgG ELISA

ELISA plates (Nunc Maxisorp) were coated with 100 µl of 2 µg/ml recombinant Vag8 diluted in carbonate buffer (15 mM Na_2_CO_3_, 35 mM NaHCO_3_ pH 9.5) for 20 h, with static incubation at 4 °C. Coated plates were washed with Tris buffered saline/Brijj buffer (0.137 M NaCl, 2.15 mM KCl, 1.1 mM Tris base, 9 mM Trizma HCl, 0.1% Brijj35 pH7.2) and blocked with 150 µl PBS containing 5% v/v foetal bovine serum and 0.1% Tween 20 for 1 h with shaking at 20 °C. Duplicate serial dilutions of the test human serum samples were diluted 1/20 in blocking buffer on a separate dilution plate. A total of 100 µl was then transferred to the coated assay plate and incubated for 2 h with shaking at 20 °C. After washing, goat anti-human IgG fragment-specific affinity purified antibody conjugated to alkaline phosphatase (Jackson Immunoresearch Laboratories), was diluted in blocking buffer 1/1000, and 100 µl applied to each well and incubated with shaking for 1 h at 20 °C. After washing the plates, 100 µl AP Yellow (p-nitrophenyl phosphate; BioFX) substrate was applied to each well and incubated for 1 h with shaking at 20 °C before the reaction was stopped by the addition of 50 µl 3 M NaOH and incubation with shaking for 5 min. The absorbance of each well was read using a Versamax plate reader (Molecular Devices) at 405 nm with reference wavelength of 690 nm. The WHO International Pertussis Standard antiserum was used as a reference serum and was assigned a value of 100 eU/ml. A 4PL curve was fitted to the data and used to interpolate ELISA concentration units from the reference curve for test sera using the SOFTmax® PRO software.

### Generation of *vag8* knockout mutant

A B1917 *vag8* knockout mutant was generated by means of homology recombination via a suicide vector. Briefly, primers were designed (Table 2) for the amplification of two sections of the B1917 genome (accession number CP009751.1). The first fragment was a 515 nucleotide region comprising the far 3’ end of *vag8* and a downstream untranslated region (2352801.2353292). The second fragment was a 531 nucleotide region internal to *vag8* (2355091.2355598). The primers were designed to insert *BsaI* sites and allow subsequent Golden Gate assembly into commercial vector pCR8, modified by cloning of a DNA fragment incorporating BsaI sites for use in Golden Gate cloning. The assembled amplified regions were then transferred into suicide vector pSS4940GW using gateway cloning. pSS4940GW is based on vector pSS4245^51^ and modified to act as a Gateway cloning^R^ destination vector (Invitrogen). The suicide vector was conjugated into B1917 as previously described^51^ using *E. coli* ST18 as the donor strain. Once in B1917, the suicide vector is unable to replicate and as such it integrates into the chromosome. Conditionally activated lethal gene *SceI* is then activated that results in excision of vector sequence and consequently in colonies that have either reverted to the wild type or that harbour the desired deletion, in this case a deletion of 1799 nucleotides within *vag8*. A representative clone harbouring the deletion, B1917Δ*vag8*, was used in these studies.

**Table 2:** Primers

Primer	Sequence
vag8-L-FW	aaaaggtctcgAACTAGGGCAGGCTGTACGAAGAC
vag8-L-RV	aaaaggtctccACATCCAATGGCAATATCGTTGAA
vag8-R-FW	aaaaggtctccATGTCATGCCTTCCTGCACATAGA
vag8-R-RV	aaaaggtctctCGAGGCACGGTATCAACGTGACTG

### Statistics

Statistical significance in deposition of complement components was calculated using two-sample *T*-test. A significant difference of *p* < 0.001 is represented by double asterisks (**) and a significant difference of *p* < 0.05 is represented by an asterisk (*). Statistical significance in ELISA was established using Mann–Whitney test. Pearson’s correlation coefficient has been calculated to measure the correlation between two variables.
